# The Correlation between Transperineal Shear-Wave Elastography and Transabdominal Ultrasound When Assessing Pelvic Floor Function in Nulliparous Women

**DOI:** 10.3390/diagnostics13183002

**Published:** 2023-09-20

**Authors:** Yerim Do, Youngeun Lim, Soohyun Lee, Haneul Lee

**Affiliations:** 1 Graduate School, Department of Physical Therapy, College of Health Science, Gachon University, Incheon 21936, Republic of Korea; doyr62@gachon.ac.kr (Y.D.); ejdnrej618@gachon.ac.kr (Y.L.); 2 Department of Physical Therapy, College of Health Science, Gachon University, Incheon 21936, Republic of Korea; pui7618@gmail.com

**Keywords:** bladder base displacement, pelvic floor muscle, shear-wave elastography, ultrasonography

## Abstract

Pelvic floor muscles (PFMs) play a crucial role in maintaining pelvic organ support and continence. However, pelvic floor dysfunction (PFD), often resulting from insufficient PFM control, poses a substantial global health challenge for women. This study aims to explore the relationship between levator ani muscle elasticity when assessed through transperineal shear-wave elastography (SWE) and bladder base displacement, quantified using transabdominal ultrasonography (TAUS), as a means to comprehensively evaluate PFM function. A total of 42 nulliparous women participated in this study. Participants received instructions on proper PFM contractions using Kegel exercises. Levator ani muscle elasticity was assessed both at rest and during contractions using transperineal SWE, while bladder base displacement was simultaneously measured through TAUS. Repeated measures demonstrated strong intraclass correlation coefficients of 0.906 at rest and 0.687 during contractions for levator ani muscle elasticity. The mean elasticity values were 24.7 ± 4.5 kPa at rest and 62.1 ± 10.4 kPa during contractions. Additionally, the mean bladder base displacement was 7.2 ± 2.5 mm, and the normalized bladder base displacement via body mass index was 0.3 ± 0.1 mm. Significantly, a moderate correlation was identified between the PFM function, represented by the difference in levator ani elasticity during contractions and resting, and bladder base displacement (r = 0.486, *p* = 0.001). These findings underscore the potential utility of transperineal SWE as a reliable and noninvasive method to assess levator ani muscle elasticity and, consequently, PFM function. In conclusion, this study sheds light on the interplay between PFM elasticity and bladder base displacement, offering insights into PFM function assessments. The observed correlation suggests the clinical relevance of SWE in providing valuable information for treatment planning in PFD. These findings contribute to a deeper understanding of PFM dynamics, ultimately aiding in the effective management of PFD among women.

## 1. Introduction

Pelvic floor muscles (PFMs), which comprise the levator ani (LA) and coccygeus muscles, play an essential role in maintaining pelvic organ support and continence within the human body [1,2]. Beyond these functions, PFMs intricately contribute to spinal stability and lumbopelvic function through co-contraction with abdominal muscles, ensuring stability in both the thoracic and pelvic regions [3,4]. The far-reaching implications of pelvic floor dysfunction (PFD), characterized by an array of distressing symptoms such as urinary and fecal incontinence, pelvic muscle weakness, pelvic organ prolapse, pelvic pain, and sexual dysfunction, are caused by poor PFM control [5,6,7]. This is a global health concern that affects millions of women worldwide, with an estimated prevalence of 25–46% [6,8]. Consequently, it leads to enormous social costs when treating PFD for healthcare [8,9]. This is caused by changes in physical and hormone levels during pregnancy alongside PFM injuries caused by vaginal delivery, which overstretches the muscles; however, other risk factors, such as age, obesity, the mode of delivery, parity, and menopause, also contribute [1,5,8,10].

Previous research underscores the importance of PFM training in PFD management, enhancing the quality of life for women [7,11]. Therefore, a comprehensive assessment of PFM function is crucial for both preventing and managing PFD on a global scale. Several techniques have been developed to evaluate PFM function, including vaginal palpation, perineometry, electromyography, magnetic resonance imaging, and ultrasound [12]. One notable approach involves quantifying the displacement of the bladder base during PFM contractions using a transabdominal ultrasound (TAUS) image [13,14,15]. The contraction of PFMs and the associated increase in fascial tension influence the bladder’s position, reflecting the muscles’ support function [16]. Notably, previous studies have indicated that TAUS can visualize the lifting action of the pelvic floor by measuring bladder base displacement [2,17]. It can visualize the direction of the bladder base and quantify the amount of bladder base displacement as it provides visual feedback on PFM contractions to the patient [15,17,18]. It has shown that bladder base displacement measured using TAUS correlates with distal palpation [2] and vaginal squeezing pressure [17], respectively, in the evaluation of PFMs. TAUS imaging is easy to use when assessing PFM function because the method is safe, noninvasive, patient-friendly, and cost-effective [17]. It is considered that the bladder base displacement assessment, which uses TAUS during PFM contractions, can provide an alternative method when evaluating the PFM functions of certain populations that are inappropriate or undesirable to invasive vaginal assessments [15].

Emerging among dynamic elastography techniques is shear-wave elastography (SWE), which assesses the mechanical properties of biological tissues by calculating shear-wave propagation velocities [19,20]. SWE has been employed to gauge muscle mechanical properties and tissue elasticity [20,21]. It has also been used to assess the elastic properties of PFM in non-pregnant women across different positions [19]. Previous research utilizing transperineal elastography has demonstrated its effectiveness when evaluating LA elasticity before and after Kegel exercises, particularly in pelvic organ prolapse patients [22]. Although investigations have explored the elastic properties of PFM during pregnancy using SWE [10], limited attention has been given when assessing the elastic properties of LA in normal nulliparous individuals, warranting a standardized reference due to variations influenced by factors like age, race, and health state [20]. Importantly, no study has established a link between LA elasticity measured via SWE and bladder base displacement measured using TAUS when assessing the PFM function. Therefore, this study addresses this gap, aiming to explore the association between LA elasticity measured by SWE and bladder base displacement quantified through TAUS for a comprehensive PFM function assessment. We hypothesize an association between the PFM function when assessed through SWE and TAUS.

## 2. Materials and Methods

### 2.1. Study Design

This observational study was conducted in accordance with the Strengthening the Reporting of Observational Studies in Epidemiology (STROBE) statement. The study was conducted in accordance with the guidelines of the Declaration of Helsinki, and the study procedure was approved by the Institutional Review Board of Gachon University (1044396-202105-HR-106-01). All participants were fully informed of the experimental procedures, and they provided written informed consent before beginning the study.

### 2.2. Participants

Forty-five nulliparous women with no history of pregnancy aged between 18 and 35 years participated in this study. The exclusion criteria included women with an experience of pregnancy or miscarriage, a personal history of PFDs, a body mass index (BMI) <18 kg/m^2^ or higher than 25 kg/m^2^, lower back pain, pelvic pain, chronic muscular disease, connective tissue disorders, or any acute infection at the time of the experiment.

The sample size was estimated using G-power 3.1.9.4 software (University of Dusseldorf, Dusseldorf, Germany). A reasonable expectation would be to detect an effect size of 0.4 with a bivariate normal correlation model, an alpha error probability of 0.05, and 85% power [23]. A sample size of 42 was required to show statistical significance when clinical differences were at an actual power of 85.05%. After estimating a dropout rate of approximately 5%, 45 participants were recruited.

### 2.3. Procedure

Upon arrival at the laboratory, participants rested comfortably in a room at an ambient temperature to be stabilized. General characteristics (height, weight, and BMI) and grip strength were measured, and information relating to exercise habits was collected. Since the assessments were measured at rest and during maximum voluntary contractions (MVC), all participants were educated on how to contract their PFMs and were instructed to practice the MVC of PFMs three times for familiarization. Another 5 min was provided for the participants to relax. They were then instructed to maximally contract the PFM and maintain MVC for 5 s while SWE and TAUS measurements were obtained simultaneously; 30 s were provided for relaxation between each contraction to prevent muscle fatigue caused by the repeated contraction of PFMs. Each condition (rest and MVC) was performed three times for the assessments.

### 2.4. PFM Contraction

Participants were instructed to lie in the lithotomy position and relax their PFMs as much as possible [19]. Kegel exercises were used to educate the participants on how to contract their PFMs. It included an explanation of how to “contract the muscle to stop the flow of urine without using the leg or abdominal muscles” [24]. Visual feedback using the E-CUBE i7 Prestige ultrasound imaging device (Alpinion Medical System, Seoul, Republic of Korea) was provided for a better understanding of participants who had difficulties when performing PFM contractions. The participants’ abdomens were palpated by an instructor to avoid abdominal muscle co-activation [24,25]. Relevant leaflets were prepared and provided to the participants prior to contraction training.

### 2.5. Outcome Measures

#### 2.5.1. Assessment of PFM (LA) Using SWE

**Figure 1:**
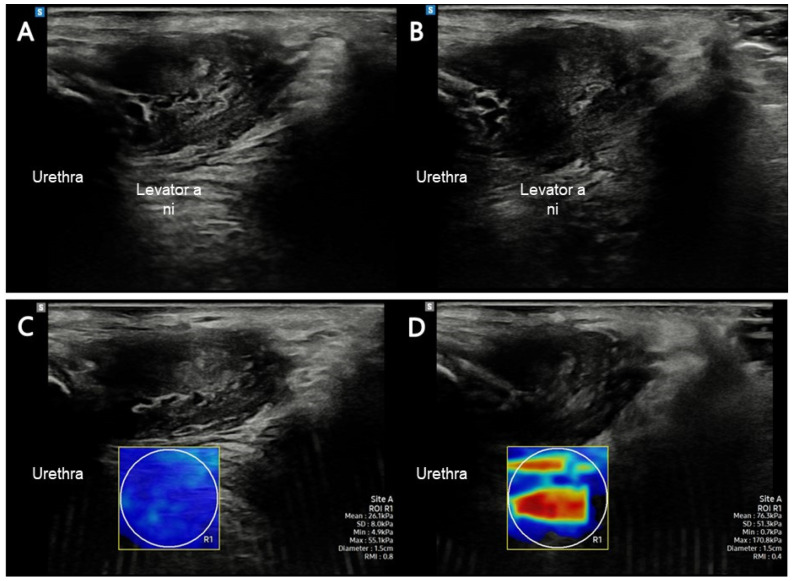
LA muscle assessment in this study. (**A**) LA muscle at rest using B-mode imaging; (**B**) LA muscle at contraction using B-mode imaging; (**C**) LA muscle at rest using shear-wave imaging; (**D**) LA muscle at contraction using shear-wave imaging. Abbreviations: LA, levator ani.

The RS85 Prestige ultrasound imaging device with a 5–10 MHz linear array transducer LA2-14A (Samsung Medicine, Seoul, Republic of Korea) was used to assess the elastic properties of the LA muscle. One experienced researcher located the transducer on the LA muscle; the probe was placed in the sagittal plane on the perineum, lateral to the vagina, at an inclination of 10° [26]. Once the researcher correctly identified the LA insertion, the participants were instructed to relax their LA muscles to obtain the SWE measurement. They were then instructed to maximally contract the LA muscle and maintain MVC for 5 s; 30 s was provided for relaxation between each contraction to prevent muscle fatigue caused by the repeated contraction. The elastic property of LA was measured three times for each condition (rest and MVC), and the mean of the three measures was considered for analysis [27]. The region of interest (ROI) was outlined manually, and two researchers were involved in selecting the video clips. We considered the minimum shear modulus in kilopascals (kPa) to assess the most relaxed state of PFM during resting and the maximum when assessing the most contracted state during MVC [27]. ROI was inserted over selected video clip images, covering the entire SWE-Box using a 1.5 cm diameter to minimize bias (Figure 1) [28].

It was required that the bladder be completely void when measuring PFMs during contraction because the sense of urination could interfere with the contraction. However, in the current study, the participants were instructed to fill their bladders with water to facilitate the simultaneous measurement of the bladder base displacement. Therefore, the LA elasticity in these two situations was also compared by measuring the state of bladder filling with water and bladder emptying in seven randomly selected participants.

#### 2.5.2. Bladder Base Displacement Using TAUS

**Figure 2:**
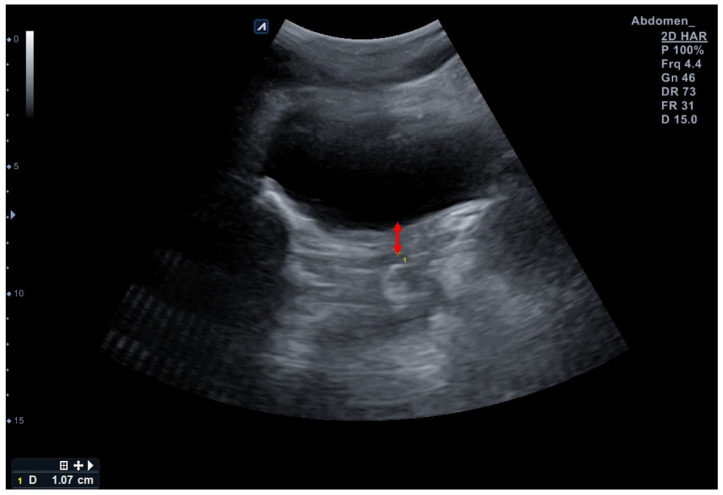
Assessment of bladder base displacement in this study using a transabdominal ultrasound. Red arrow indicates bladder base displacement during contraction.

The E-CUBE i7 Prestige ultrasound imaging device with a 4.4-MHz convex transducer C1-6T (Alpinion Medical System, Seoul, Republic of Korea) was used for the TAUS measurements. The participants were instructed to fill their bladder with 600–700 mL of water 1 h before the assessment without voiding according to a standardized bladder filling protocol for the clear imaging of the bladder base [14,29]. One researcher placed the probe transversely in the suprapubic region to find the bladder base. The probe was angled in the posterior/caudal direction, and the variety of the angles was approximately 15–30° depending on the participant’s state of bladder fullness to obtain a clear image of the bladder. Once the sonographer correctly found the bladder, the participants were instructed to relax their PFMs to obtain SWE and TAUS measurements. The probe of SWE was placed in the sagittal plane on the perineum, and the probe for TAUS was placed transversely in the suprapubic region [27]. Both measurements were used three times at the same time and the mean was considered for analysis [27]. The clearest displacement of the central part of the bladder base at the point where the hyper-hypoechoic structures met during resting, and the contraction of the PFM was expressed with two points. The distance between these two marked points (red arrow) shows the amount of elevation experienced by the bladder base during contraction; descent during resting was measured to assess the bladder base displacement using an ultrasound caliper, which was marked in millimeters (mm) (Figure 2) [14]. The value was negative if the bladder base descended despite the PFM contraction. The PFM function was considered based on the value of the bladder base displacement.

### 2.6. Statistical Analysis

The SPSS 26.0 software for Windows 10 (IBM, Armonk, NY, USA) was used to analyze data. Data are summarized using the mean and standard deviations (SDs) for the quantitative variables. The normality of continuous variables was examined using the Shapiro–Wilk test. The intraclass correlation coefficient (ICC) was calculated to assess the intra-tester reliability of these measurements. The agreement between the test and re-test measurements with a confidence level [CI] of 95% was calculated using the Bland–Altman plot. Since all outcome variables were normally distributed, a paired *t*-test was used to compare the means of elastic properties for the LA muscle during the resting position and MVC. Pearson’s correlation analysis was used to examine the relationship between the elastic properties of the LA using SWE while bladder base displacement used TAUS. The level of significance was set at *p* = 0.05.

## 3. Results

The mean age of all the participants was 23.6 ± 3.2 years, and the mean ± SD BMI was 21.5 ± 2.1 kg/m^2^. Among the 45 participants, three (6.6%) failed to demonstrate a correct PFM contraction when conducting the measurement; therefore, three participants were excluded from the statistical analysis. Their anthropometric data were not significantly different from others (mean age of 24.7 ± 1.5 years and BMI of 21.4 ± 1.6). The general characteristics of the participants are presented in Table 1.

**Figure 3:**
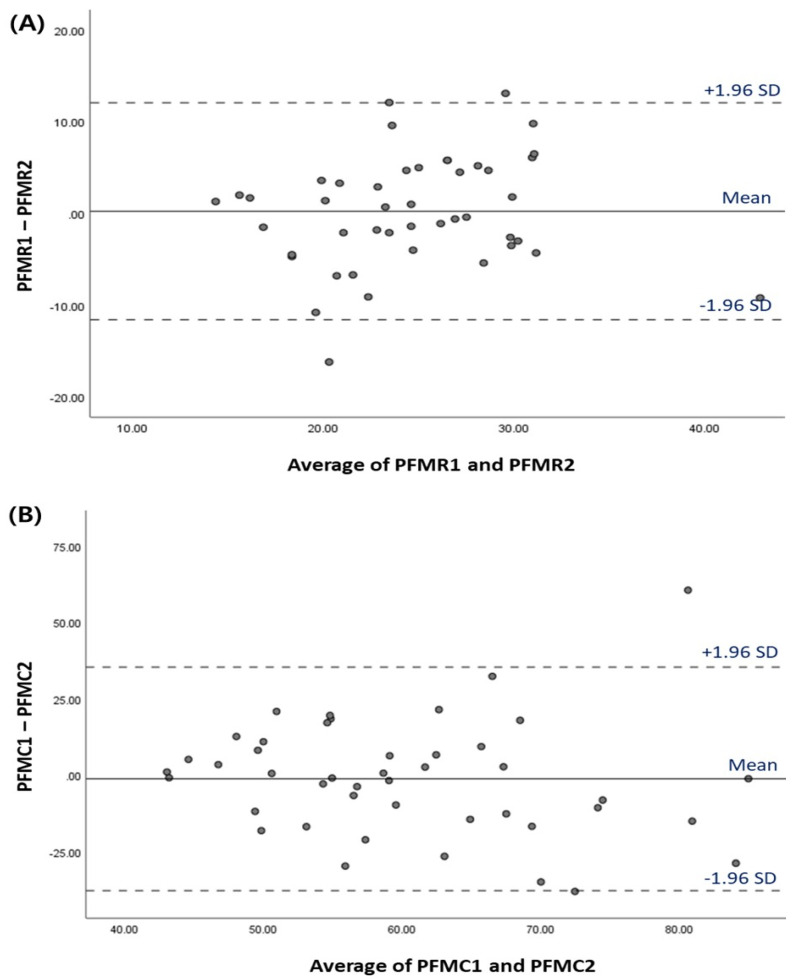
Bland–Altman plot for LA muscle elasticity measurements via SWE between the test and re-test (**A**) Resting (**B**) Contraction. The means of the test and re-test scores is plotted on the X-axis, and the differences between these two scores is shown on the Y-axis. Abbreviations: PFMR, pelvic floor muscle elasticity at resting; PFMC, pelvic floor muscle elasticity during contraction.

The ICC_(2,1)_ for the repeat measures of LA muscle elasticity at rest and contraction was moderate to excellent (0.906 at rest and 0.687 during contraction). The Bland–Altman plot of agreement for PFMs during resting and contraction measurements via SWE between the test and re-test is shown in Figure 3. The Bland–Altman plot showed that 95% of data were within the limits of agreement for the test and re-test.

The LA muscle elasticity measured via SWE was 24.7 ± 4.5 kPa at rest and 62.1 ± 10.4 kPa during contraction. There was a significant increase in the mean LA muscle elasticity when the muscle voluntarily contracted compared to resting (95% CI: 34.3–40.4, *p* < 0.001, Table 2). Also, there was no significant difference in LA muscle elasticity between the state of bladder filling and emptying both at rest and during contraction (resting, 24.8 ± 2.5 vs. 26.1 ± 2.9; contraction, 57.9 ± 11.9 vs. 58.1 ± 13.4; *p* > 0.05).

The mean bladder base displacement of the participants was 7.2 ± 2.5 mm (Table 2), and the normalized bladder base displacement using BMI was 0.3 ± 0.1 mm.

**Figure 4:**
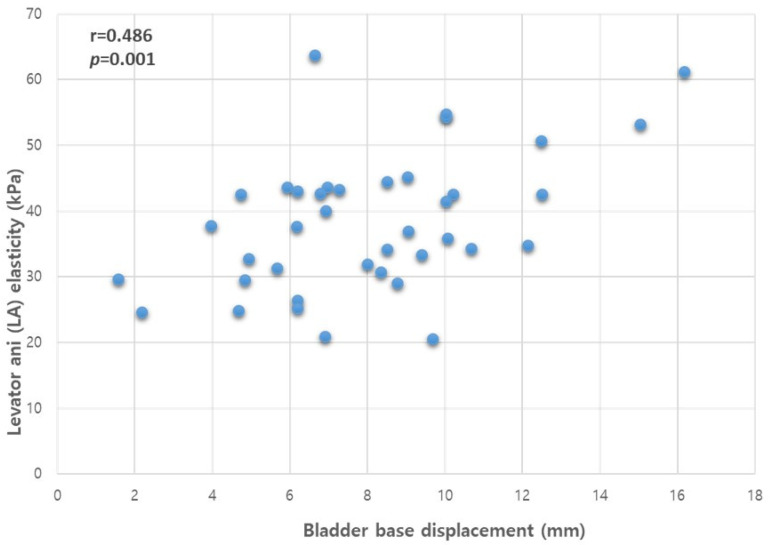
Correlation between the bladder base displacement and LA muscle elasticity. Abbreviations: LA, levator ani; r, Pearson’s correlation coefficient.

Pearson’s correlation analysis revealed a statistically significant association between bladder base displacement and the differences in PFM elasticity between contraction and resting states when assessed concurrently (r = 0.486, *p* = 0.001, Figure 4).

## 4. Discussion

PFDs are recognized as a global health concern, profoundly impacting millions of women across the world. With a growing elderly population, the burden of PFDs is poised to escalate, warranting heightened attention and intervention [30]. While surgical approaches to pelvic organ prolapse offer structural restoration, functional outcomes often leave much to be desired, prompting a shift toward analyzing the biomechanical intricacies underlying these conditions [31]. As these problems are structural failures, researchers have begun to analyze the mechanisms of theis biomechanical system [31] and have found that PFM training and surgery play an important role in PFM function [32]. A recent systematic review indicated the efficacy of PFM training in bolstering strength and endurance, surpassing control interventions [32]. However, limited evidence exists regarding early preventive measures using noninvasive tools to measure the elastic properties of LA muscle values when assessing PFM function. Implementing early preventive programs by assessing the elasticity of LA muscle function compared with normative values is critical for preventing PFDs. Moreover, no study has assessed the correlation between LA muscle elasticity and bladder base displacement using noninvasive methods for PFM function assessment. Therefore, to our knowledge, this is the first observational study to assess the elastic properties of the LA muscle in nulliparous women and assess bladder base displacement using TAUS. We investigated the association between LA muscle elasticity using SWE and bladder base displacement via TAUS to evaluate PFM function. Our results revealed a moderate correlation coefficient between LA elasticity, assessed noninvasively via SWE, and bladder base displacement measured with TAUS: a gold standard technique that indirectly measures PFM function [33]. We observed excellent reproducibility at rest (ICC_(2,1)_ = 0.906) and moderate reproducibility during contraction (ICC_(2,1)_ = 0.687), reinforcing the robustness of our measurements.

Several studies have assessed PFM function using direct and indirect methods; however, our results are difficult to compare with those of previous studies because of different metric values [14,15,27]. Comparative analysis remains challenging due to the distinct nature of the metrics used in previous investigations, rendering direct comparisons intricate. Unlike prior cadaveric research, which is focused on damage thresholds, our focus on intrinsic elastic properties sets our study apart [20,29,34]. Notably, the results recorded by the force sensor of the vaginal speculum measuring the amount of force applied on the probe are not direct quantitative evaluations of elastic properties such as those using elastography [20]. Interestingly, our results align with Gachon et al., who reported comparable shear modulus values for LA muscle elasticity. They reported a shear modulus of 21.9–22.8 and 55.1–61.4 kPa at rest and during contraction, respectively, while our results revealed an average of 24.7 and 62.1 kPa at rest and during contraction, respectively, indicating that our results are similar [20]. Bladder base displacement has also been assessed in previous studies conducted in specific or mixed populations [2,15,29]. As our study only included nulliparous women with no history of pregnancy, bladder base displacement is similar to that reported by Sherburn et al., which included premenopausal nulliparous women as their participants [35], and the normalized value obtained when dividing bladder base displacement by BMI is similar to that of Iman et al. [29]. Additionally, our results reveal a stronger ICC for repeated measures of PFM when the LA muscle is at rest compared to during contraction, which supports the results of a previous study [20]. This could be explained by the lower detection accuracy of SWE measurements owing to the generation of high-speed shear waves that are too fast to detect when assessing strongly contracted muscles [36].

This study demonstrates SWE to be a reliable tool for investigating the elastic properties of PFM in nulliparous women. Additionally, this study adds important findings in terms of establishing an association between bladder base displacement and LA elasticity differences between contraction and resting for the pelvic floor function and providing a standard reference for LA muscle elasticity to assess pelvic floor function in nulliparous women. This reference might help with routine pelvic floor assessments and the prevention and management of PFDs. An appropriate assessment of the PFM function is important when applying a PFM strengthening intervention and determining the proper strengthening load. Understanding how to correctly perform PFM contractions might be helpful for patients who need PFM strengthening exercises, and it could also improve their quality of life [11,29].

Although this is the first study to assess the correlation between bladder base displacement and LA elasticity differences between contraction and resting, certain limitations warrant acknowledgment. First, although we assessed LA muscle elasticity in the lithotomy position with both an empty and a filled bladder to establish that these two statuses do not affect LA muscle elasticity, the state of the bladder, when filled with water might interfere with the contraction intensity, which was used as the data of bladder base displacement. Second, active voluntary contraction was not measured quantitatively using electromyography or clinically using vaginal palpation. Third, the pubic symphysis was used as a starting point to measure bladder base displacement, indicating that there was no bony landmark within sight. This could cause a movable starting point because it can only be potentially expressed. This study included only young nulliparous women. Future studies should include different age groups or childbirth statuses (nulliparous and multiparous) to establish a proper standard reference for LA muscle elasticity and assess pelvic floor function.

## 5. Conclusions

In summary, our findings establish a moderate correlation between PFM function, as indicated by variations in LA elasticity during contractions and resting, and bladder base displacement. Furthermore, our study underscores the potential of SWE technology to assess LA elasticity non-invasively, offering a novel avenue for comprehensive PFM function evaluation. This promising technique holds the potential to inform tailored treatment strategies for PFD, ushering in a new era of improved clinical management.

## Figures and Tables

**Table 1:** General characteristics of participants.

	Nulliparous Women (*n* = 42)
Age (years)	23.5 ± 3.2
Height (cm)	162.4 ± 4.6
Weight (kg)	56.7 ± 6.7
BMI (kg/m^2^)	21.5 ± 2.1
Grip strength (kg)	26.8 ± 4.3

Abbreviation: BMI, body mass index.

**Table 2:** LA muscle elasticity and bladder base displacement for PFM function in nulliparous women (*n* = 42).

	Rest	Contraction	95% CI
LA elasticity (kPa)	24.7 ± 4.5	62.1 ± 10.4 *	34.3–40.4
Bladder base displacement (mm)	7.2 ± 2.5		-

Abbreviation: LA, levator ani; PFM, pelvic floor muscle; CI, confidence interval. * Significant difference between rest and contraction (*p* < 0.001).

## Data Availability

The datasets generated in this study are available from the corresponding author upon reasonable request.
